# Sequence similarity between the erythrocyte binding domain 1 of the *Plasmodium vivax *Duffy binding protein and the V3 loop of HIV-1 strain MN reveals binding residues for the Duffy Antigen Receptor for Chemokines

**DOI:** 10.1186/1743-422X-8-45

**Published:** 2011-01-31

**Authors:** Michael J Bolton, Robert F Garry

**Affiliations:** 1 Vaccine and Infectious Disease Institute, Fred Hutchinson Cancer Research Center Division of Allergy and Infectious Diseases University of Washington 1100 Fairview Avenue Seattle, Washington 98109 USA; 2 Department of Microbiology and Immunology Tulane University 1430 Tulane Avenue New Orleans, Louisiana 70112 USA

## Abstract

**Background:**

The surface glycoprotein (SU, gp120) of the human immunodeficiency virus (HIV) must bind to a chemokine receptor, CCR5 or CXCR4, to invade CD4+ cells. *Plasmodium vivax *uses the Duffy Binding Protein (DBP) to bind the Duffy Antigen Receptor for Chemokines (DARC) and invade reticulocytes.

**Results:**

Variable loop 3 (V3) of HIV-1 SU and domain 1 of the *Plasmodium vivax *DBP share a sequence similarity. The site of amino acid sequence similarity was necessary, but not sufficient, for DARC binding and contained a consensus heparin binding site essential for DARC binding. Both HIV-1 and *P. vivax *can be blocked from binding to their chemokine receptors by the chemokine, RANTES and its analog AOP-RANTES. Site directed mutagenesis of the heparin binding motif in members of the DBP family, the *P. knowlesi *alpha, beta and gamma proteins abrogated their binding to erythrocytes. Positively charged residues within domain 1 are required for binding of *P. vivax *and *P. knowlesi *erythrocyte binding proteins.

**Conclusion:**

A heparin binding site motif in members of the DBP family may form part of a conserved erythrocyte receptor binding pocket.

## Introduction

Human immunodeficiency virus type 1 (HIV-1) and the human malaria parasite *Plasmodium vivax *both use chemokine receptors in obligate steps of cell invasion. HIV-1 uses CCR5 and CXCR4 as the major coreceptors for infecting CD4+ cells (macrophages, T-lymphocytes, and other cell types) *in vivo*, while *P. vivax *uses the Duffy antigen receptor for chemokines (DARC) for invading human reticulocytes [1,2]. Alleles of CCR5 and DARC associated with decreased functional protein expression confer resistance to HIV and *P. vivax*, respectively, and chemokines can inhibit *in vitro *infection by either pathogen [1,3-5]. The HIV surface glycoprotein (SU, gp120) undergoes a conformational change upon binding to CD4 and then presents a chemokine receptor binding surface predicted to include a hydrophobic core surrounded by positive residues contributed by conserved and variable regions including the base of the V3 loop. The V3 loop putatively extends toward the cell surface and contacts the chemokine receptor at a second site in the second extracellular loop. Individual amino acid mutations in the V3 loop can change chemokine receptor specificity.

*P. vivax *and the simian malaria, *P. knowlesi*, use Duffy binding proteins (PvDBP and PkDBP) to invade human erythrocytes. These proteins belong to a family of erythrocyte binding proteins with conserved regions. The erythrocyte binding domains of PvDBP and PkDBP (or *P. knowlesi α *protein) have been shown to map to the 330 amino-acid cysteine-rich region II known as the Duffy-binding-like (DBL) domains [6]. Other members of the family include the *P. knowlesi β *and *γ *proteins and the *P. falciparum *erythrocyte-binding antigen (EBA-175), which use DBLs to bind to other receptors.

Here we report the identification of an amino acid sequence similarity between the V3 loop of HIV-1 strain MN and a site in *Plasmodium *erythrocyte binding proteins that contains a consensus heparin binding site. Both HIV-1 and *P. vivax *can be blocked from binding to their chemokine receptors by the chemokine RANTES. Mutagenesis studies suggest that the heparin binding site motif in members of the DBP family may form part of a conserved erythrocyte receptor binding pocket.

## Materials and methods

### Sequence comparisons

William Pearson's LALIGN program, which implements a linear-space local similarity algorithm, was used to perform regional alignments. Sequence and structural comparisons were performed for the V3 loop of SU of HIV-1 strain MN, accession: AAT67509; *P. vivax *DBP, ACD76813; *P. knowlesi *DBP, XP_002261904; *P. falciparum *erythrocyte binding protein EBA-175 (F1), accession AAA29600. *Plasmodium *proteins are members of pfam05424 (a member of the superfamily cl05146).

### Erythrocytes

Blood was collected in 10% citrate phosphate dextrose (CPD) and stored at 4°C unwashed for up to 4 weeks, or washed in RPMI with malaria supplements and stored in malaria culture medium at 50% hematocrit for up to 2 weeks. The DARC+ human erythrocytes used in the erythrocyte binding assay and the *P. knowlesi *erythrocyte invasion assay had the phenotype Fy(a^-^b^+^) as determined by standard blood banking methods using anti-Fya and anti-Fyb antisera (Gamma Biologicals, Houston, TX). Erythrocytes were washed three times in DMEM (Gibco BRL) and resuspended to a hematocrit of 10% in complete DMEM for the erythrocyte binding assay. Erythrocytes used in the *P. knowlesi *erythrocyte invasion assay were washed three times and resuspended to a hematocrit of 10% using malaria complete RPMI.

### Cell Culture and Transfection of COS-7 Cells

COS-7 cells (ATCC CRL 1651; Rockville, MD) were cultured in DMEM with 10% heat inactivated FBS (Gibco BRL) in a humidified 5% CO_2 _incubator at 37°C. Cells were seeded in polystyrene dishes with 3.5-cm diameter wells and grown for 24 h to 30-50% confluence before transfection with 1 mg of pHVDR22 plasmid DNA and 10 ml of Lipofectamine (Gibco BRL).

### P. knowlesi in vitro culture

Whole blood from rhesus macaques was collected in 10% CPD and allowed to separate overnight at 4°C. The erythrocyte phase was washed in RPMI with L-glutamine and supplemented with 25 mM HEPES, 300 mM hypoxanthine, 10 mM thymidine, 1.0 mM sodium pyruvate, and 11 mM glucose. This RPMI with malaria supplements was then used to prepare malaria culture medium by adding to a final concentration of 0.24% sodium bicarbonate and 0.2% Albumax-I (Life Tech, Gibco BRL). Cultures were maintained at a hematocrit of 10% in malaria culture medium under an atmosphere of 5% O_2_, 5% CO_2_, balanced N_2 _(Air Liquide, Houston, TX) at 38°C.

### Percoll Purification of Schizont-infected Erythrocytes

Cultures of *P. knowlesi *at 5-10% infected erythrocytes were washed three times in RPMI with malaria supplements and 10% FBS and brought up to a hematocrit of 10%. A 50% Percoll solution was made by adding 0.45 volumes 1X PBS, 0.05 volumes 10X PBS and 0.5 volumes Percoll (Sigma). Two ml of the washed culture was overlaid on 2 ml of the 50% Percoll solution in a 4 ml polystyrene tube and centrifuged for 20 min at 2100 RPM in a Sorvall centrifuge. The ring of cells at the interface was removed, pooled and washed three time in 1X PBS. The pellet was brought up in malaria culture medium to 2 × 10^7 ^cells/ml.

### PvRII Erythrocyte Binding Assay

COS-7 cells were transfected by Lipofectamine with 1-2 mg of pHVDR22 DNA, a plasmid kindly provided by L. Miller which expresses region II of the DBP of P. vivax on the cell surface as a chimera with the HSV gD protein [7] Duffy Fy (a-b+) erythrocytes were washed three times in RPMI 1640, resuspended to a hematocrit of 1% in 1 ml of complete DMEM with the chemokines RANTES, MIP-1α, SDF-1 or AOP-RANTES at concentrations of 0, 0.1, 1, 10, and 100 nM for 1 h at 37°C (Peprotech, Gryphon Pharmaceuticals, San Francisco, CA). This suspension was swirled over aspirated COS-7 cells 40-60 h after transfection and allowed to settle over 2 h at 37°C. The COS-7 cells were then washed three times with 2 ml of PBS to remove nonadherent erythrocytes. The number of adherent erythrocyte rosettes was scored in 20 randomly chosen fields at a magnification of 40 using an inverted microscope. Percent inhibition was determined by dividing the number of rosettes in the presence of chemokines by the number at a concentration of 0 nM. The 50% inhibitory concentration (IC_50_) was determined by the mean of three separate experiments to use in a semi-log cubic spline curve fit with the DeltaSoft 3 software (Biometallics, Inc., Princeton, NJ).

### P. knowlesi Erythrocyte Invasion Assay

Human Duffy Fy(a^-^b^+^) erythrocytes were washed in complete malaria medium and 2 × 10^7 ^washed cells were added to increasing concentrations of chemokines in malaria culture medium at final volume of 900 ml for 1h at room temperature. To each tube of chemokine-treated erythrocytes, 100 ml or 2 × 10^6 ^schizont-infected erythrocytes was added and placed in a well of a polystyrene 24-well plate (Becton-Dickinson). The cultures were maintained under a blood-gas atmosphere at 38°C for 8 h to allow the infected erythrocytes to rupture and release free merozoites capable of infecting new erythrocytes and developing to ring-stage trophozoites. The culture was centrifuged at 2100 RPM for 3 min and a thin smear was made from the pellet. The thin smear was fixed with methanol and stained with Leukostat Solution B (100 mg Eosin Y+300 ml 37% formaldehyde + 400 mg sodium phosphate dibasic + 500 mg potassium phosphate monobasic, q.s. to 100 ml with dH_2_O), rinsed, and stained with Leukostat Solution C (47 mg Methylene Blue + 44 mpp Azure A + 400 mg sodium phosphate dibasic + 500 mg potassium phosphate monobasic, q.s to 100 ml with dH_2_O). The percentage of erythrocytes infected with ring-stage trophozoites per 2000 erythrocytes was determined at 1000X. Inhibition of invasion expressed as % inhibition was determined by dividing the percentage of ring-stage parasites by the percentage of ring-stage parasites at 0 nM chemokine, multiplying by 100 and subtracting this value from 100 [1].

### Statistical analysis

The software StatView (Brainpower, Inc., Calabasas, CA), was used to determine the statistical difference between the inhibitory concentrations of RANTES, AOP-RANTES, and MIP-1α, using a two-way ANOVA test.

### Plasmids

The plasmids pHVDR22, pHKADR22, pHKBDR22 and pHKGDR22 encode for the region II (amino acids 198-522) of the *P. vivax *DBP and region II of the *P. knowlesi *α, β and γ genes, respectively, in the context of the HSV gD protein. These plasmids have been previously described and were kindly provided by the laboratory of Louis H. Miller. These plasmids were created from the plasmid pRE4, which contains an SV40 origin of replication, a Rous sarcoma virus LTR as a promoter, the coding region of the HSV glycoprotein D (HSV *gD*) inserted in the HindIII cloning site, and the SV40 early polyadenylation signal. The HSV *gD *features a 25 amino acid signal peptide at the amino terminus, a 24 amino acid hydrophobic transmembrane region, a 30 amino acid cytoplasmic tail at the carboxy terminus, and two epitopes at amino acids 11-19 and 272-279 that can be targeted specifically with the monoclonal antibodies ID3 and DL6, respectively. The region II sequences were inserted between the unique Apa I and Pvu II restriction sites.

### Cloning and Site Directed Mutagenesis

Mutants of the region II expressing plasmids were generated by three strategies: inverse PCR, PCR and restriction digestion, or PCR-based site directed mutagenesis. Each mutant was sequenced by Research Genetics, Inc. (Huntsville, Ala.) to confirm proper construction at the site of mutation.

The following constructs were made from the pHVDR22 plasmid:

*pv22d32 *This construct contains a deletion in amino acids 216-247 of the RII of *P. vivax*, which corresponds to the V3-like peptide region with similarity to the V3 loop and comprises cysteines C1 to C4 of region II. For lack of proper restriction enzyme sites, an inverse PCR strategy was use to amplify the entire pHVDR22 plasmid flanking the site to be deleted. The primers 5'TGT ATG AAG GAA CTT ACG AAT TTG G3' and 5'TTT CAT TAC AGT ATT TTG AAG3' were first phosphorylated with T_4 _kinase then used with the long range, high fidelity DeepVent polymerase (New England Biolabs, Inc., Beverly, MA) to amplify the product under the following thermocycling conditions: 5 minutes at 94°C initial denaturing, then 35 cycles at 94°C for 60 seconds, 55°C for 60 seconds, 72°C for 3 minutes. The product was digested with DPN I to eliminate methylated input plasmid DNA, then blunt-end ligated with high concentration ligase (Gibco BRL).

*pv22MNV3 *This construct replaces the 32 amino acid V3-like peptide of the *P. vivax *RII with the V3 loop of HIV-1 strain MN. To amplify the V3 loop of HIV-1_MN _by PCR, PM-1 cells were infected with HIV-1_MN _(donated by Dr. James Robinson, Tulane University Medical Center) and genomic DNA was isolated from infected cultures. This DNA includes proviral DNA and was used as template for a PCR with the primers P2 5'GAC GCT GCG CCC ATA GTG CTT CCT G3' and P5 5'ACA CAT GGAATT CGGCCAGTA GT3' which are homologous to conserved regions of the *env *gene of HIV and amplify the region between nucleotides 6884 and 7783, which includes the V3 loop.

This PCR product then served as template in a nested PCR of the HIV-1_MN _V3 loop using the primers HVMN-F 5'AATTGTACAAGACCCAACTAC3' and HVMN-r 5'ATGTGCTTGTCTTATAGTTCC3'. This nested PCR was carried out using the DeepVent enzyme to generate blunt ends. The product of the second, nested PCR was gel purified. The gel-purified amplicon was then re-amplified in a 300 ml PCR using HVMN-r and HVMN-F primers, which were first phosphorylated with T_4 _poly N kinase. This reamplification product was column purified and blunt-end ligated to the inverse PCR product described in the preparation of pvD32. The sequenced construct matched the MN V3 sequence as previously published.

*pv22suf32 *This construct was designed to determine if the 32-aa V3-like peptide of *P. vivax *RII is sufficient for DARC binding by deleting all flanking RII amino acids. The primers used to create this construct were 5'CAA AAT CAG CTG ATG AAA AAC TGT AAT TAT3' and 5'CAA ATT GGG CCC TTC CTT CAT ACA TAA TTG3' and contain the restriction sites for Apa I and Pvu II. The pHVDR22 plasmid was digested with Apa I and Pvu II, and the digested vector was separated from the insert by gel electrophoresis and extracted using the QIAEX II gel extraction kit (Qiagen Inc., Valencia, CA). The PCR product was also digested with Apa I and Pvu II and ligated to the digested vector.

*pv22d5C1 *This construct deletes amino acids 198-216, or the 5' flanking region to C1. This was created using the primers 5'TGT ATG AAG GAA CTT ACG AAT TTG G3' and 5' GGG GCC TTG GGC CCT GTC ACA AC3', the product of which was digested with Apa I and Pvu II and cloned into the digested vector as described for psuf32

*pv22d3C4 *This construct deletes amino acids 247 to 522 or the 3' flanking region to C4. This was created using the primers 5'CCG GTC CTG GAC CAG CTG ACG3' and 5'TTT CAT TAC AGT ATT TTG AAG3' the product of which was digested with Apa I and Pvu II and cloned into the digested vector as described in psuf32

*pv22d5C4 *This construct deletes amino acids 198 to 247 or the 5' flanking region to C4 This was created using the primers 5'CAA TTA CAG CTG AAG GAA CTT ACG AAT TTG3' and 5' GGG GCC TTG GGC CCT GTC ACA AC3' the product of which was digested with Apa I and Pvu II and cloned into the digested vector as described in pv22suf32

*pv22KARA *The Stratagene QuickChange kit (Promega) was used to mutate the heparin binding consensus site in PvRII at amino acids 217-226 from YKRKRRERDW to YARKAREADW using the primers 5' GTA ATT ATG CGA GAA AAG CTC GGG AAG CAG ATT GG3' and 5' CCA ATC TGC TTC CCG AGC TTT TCT CGC ATA ATT AC3'. These primers also introduce an Ava I site as a silent mutation for screening.

*pv22KAKA *The Stratagene QuickChange kit was used to mutate a second potential heparin binding consensus site at amino acids 364-373, between C5 and C6, from SVKKRLKGNF to SVKARLAGNF using the primers 5' GAT GTA CTC AGT TAA AGC AAG ACT TAA GGG G3'. These primers also introduce an Afl II site as a silent mutation for screening

*pv22KA *The Stratagene QuickChange kit was used to introduce a single alanine substitution in the heparin binding consensus site at amino acids 217-226 from YKRKRRERDW to YKRARRERDW. 5'CTC TTT CCC GAC GAG CTC TCT TAT AAT TAC AG3' and 5'CTG TAA TTA TAA GAG AGC TCG TCG GGA AAG AG3'. These primers also introduce a Sac I site as a silent mutation for screening.

The following mutants were made from the pHKADR22, pHKBDR22, or pHKGDR22 plasmids using the Stratagene QuickChange kit:

*pkalpha22KARA *This mutant was designed to change the heparin binding consensus site in pHKADR22 at amino acids 217-226 from DKRKRGERD to DARKAGEAD using the primers 5'GTC CCA ATC TGC TTC CCC GCG AGC TCT CGC ACT ACC ACA CTT G and 5'CAA GCG TAA TGA TGC GAG AGC TCG CGG GGA AGC AGA TTG GGA C3'. These primers also introduce a Sac I site as a silent mutation for screening.

*pkbeta22KARA *This mutant was designed to change the heparin binding consensus site in pHKBDR22 at amino acids 217-226 from NKRKRGTRD to NARKAGTAD using the primers 5' CAG TCC CAA TCT GCT GTC CCG CGA GCT TCT GCA TTA TTA CAC C3' and 5'GGT GTA ATA ATG CGA GAG CTC GCG GGA CAG CAG ATT GGG ACT G3'. These primers also introduce a Sac I site as a silent mutation for screening.

*pkgamma22KARA *This mutant was designed to change the heparin binding consensus site in pHKGDR22 at amino acids 217-226 from DKRKRGERD to DARKAGEAD using the primers 5'GTC CCA ATC TGC TTC CCC GCG AGC TCT CGC ACT ACC ACA CTT G and 5'CAA GCG TAA TGA TGC GAG AGC TCG CGG GGA AGC AGA TTG GGA C3'. These primers also introduce a Sac I site as a silent mutation for screening.

### Immunofluorescence Staining

Transfected cells used in the erythrocyte binding assay were rinsed in PBS and incubated for 1 h at 37°C with monoclonal antibodies that bind to amino acids 11-19 and 272-279 of the mature HSV gD protein found in pHVDR22. These primary antibodies, ID3 or DL6 (provided by Drs. Gary Cohen and Roselyn Eisenberg), were used at a 1:2000 dilution in PBS containing 10% FBS. The cells were rinsed with PBS and incubated at 37°C with fluorescein conjugated anti-mouse antibodies at 1:100 in PBS containing 10% FBS. Untransfected COS-7 cells were also stained as a negative staining control. The cells were then fixed with 4% paraformaldehyde for 15 min, and observed for surface expression of the products of the transfected plasmids using an inverted fluorescence microscope.

## Results

### Homologous sequences in Plasmodium erythrocyte binding proteins and the V3 loop of HIV-1 SU

**Figure 1:**
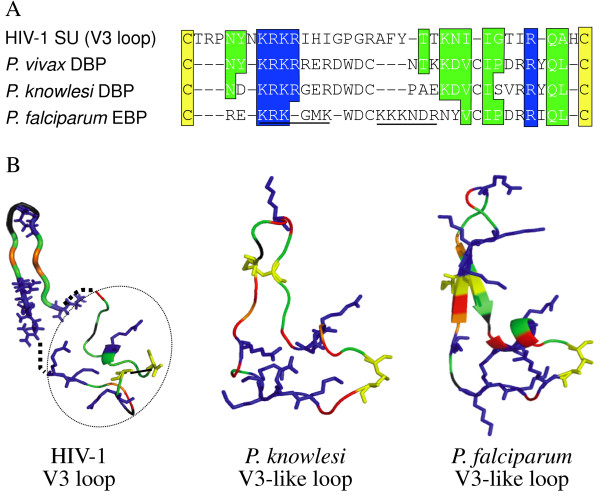
**Similarities between peptides in the V3 loop of HIV-1 and conserved Plasmodium erythrocyte binding proteins**. Panel A: Homologous sequences in the cysteine-rich regions of the *P. falciparum *erythrocyte binding protein EBA-175 (F1*), P. knowlesi *DBP (a), *P. vivax *DBP, and the V3 loop of HIV-1 strain MN. Identical or similar amino acids are boxed in yellow or green in both panels. KRKR (in green box) is a consensus glycosaminoglycan (GAG) binding sequence (BBXB, where B is a basic amino acid, K or R) in the HIV-1, *P. vivax *and *P. knowlesi *sequences. *P. falciparum *EBA-175 has two GAG binding sites of the BBBxxB type in tandem (underlined). Panel B: Secondary structure of the V3 loop of HIV-1 SU strain MN as determined by Sharon et al. [14] is shown (PDB: 1NJ0). Addition residues not contained in this structure were modeled in SWISS-MODEL (dashed oval). Structures are shown for the V3 loop-like regions of *P. knowlesi *DBP as determined by Singh et al. [10] (2C6J) and *P. falciparum *EBA-175 as determined by Tolia et al. [15] (1ZRL).

The common use of chemokine receptors by HIV-1 SU and PvDBP suggested the possibility that these proteins may share structural or functional motifs. A homology search lead to identification of an amino-acid sequence similarity between the V3 loop of HIV-1 strain MN and a 32-aa site within region II of PvDVP, which maps to domain 1 (Figure 1). Homologous sequences are present in the cysteine-rich regions of the *P. falciparum *erythrocyte binding protein EBA-175 (F1*) and P. knowlesi *DBP. There is a consensus glycosaminoglycan (GAG) binding sequence (BBXB, where B is a basic amino acid, K or R) in the HIV-1 MN, *P. vivax *and *P. knowlesi *sequences. *P. falciparum *EBA-175 has two GAG binding sites of the BBBxxB type in tandem.

### Blocking of PvRII binding to DARC by RANTES and AOP-RANTES

**Figure 2:**
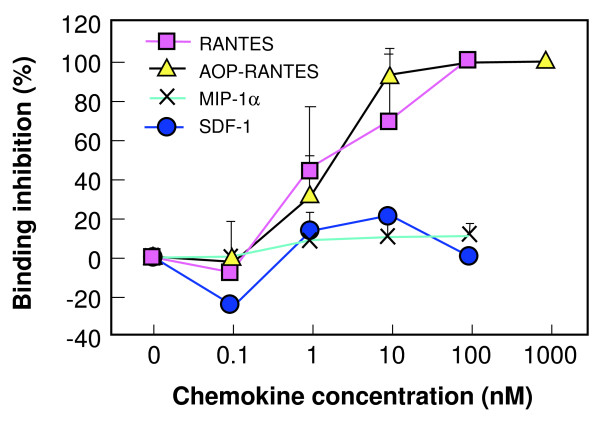
**Chemokine inhibition of PvRII binding to DARC+ erythrocytes**. The erythrocyte rosette assay of Chitnis and Miller [6] was used to quantify chemokine inhibition of PvRII Binding to DARC+ Erythrocytes. Binding was determined by subtracting the number of COS-7 cells expressing pvRII with rosettes of chemokine-treated DARC+ human erythrocytes (per 20 fields at 200X magnification) from the number with rosettes of untreated erythrocytes, and dividing by the number with rosettes of untreated erythrocytes. The data shown are the mean of three separate experiments.

The erythrocyte binding assay of Chitnis and Miller [6] was used to determine the inhibitory concentrations of chemokines for region II of the *P. vivax *DBP binding to DARC. Both RANTES and AOP-RANTES elicited a dose-response inhibition of binding (Figure 2). MIP-1α is known not to bind to DARC, and was included as a negative control. SDF-1, the natural ligand of CXCR-4 and an inhibitor of X4 viruses, has not been tested for DARC binding in the published literature, and did not inhibit binding in the erythrocyte binding assay. The IC_50 _for RANTES and AOP-RANTES were 2.09 nM and 1.51 nM, respectively. The difference in response as determined by a two-way ANOVA test was significant between RANTES and MIP-1α and between AOP-RANTES and MIP-1α, but not between RANTES and AOP-RANTES (p < 0.05).

### Blocking of P. knowlesi invasion of DARC+ human erythrocytes by RANTES and AOP-RANTES

**Figure 3:**
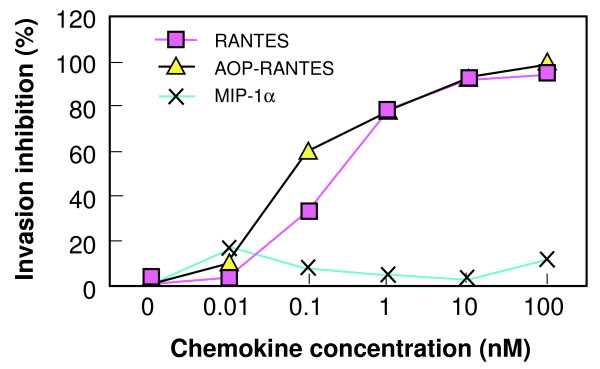
**Chemokine inhibition of *P. knowlesi *invasion of DARC+ erythrocytes**. Inhibition of *P. knowlesi *invasion of DARC+ erythrocytes was determined by subtracting the number of chemokine-treated DARC+ human erythrocytes invaded by *P. knowlesi *merozoites (per 2000 erythrocytes) from the number of untreated DARC+ human erythrocytes invaded by *P. knowlesi *merozoites, and dividing by the number of untreated, invaded erythrocytes.

A standard erythrocyte invasion assay was used to determine the chemokine inhibitory concentrations of DARC-dependent invasion of human DARC+ erythrocytes by *P. knowlesi*. Both RANTES and AOP-RANTES elicited a dose-response inhibition of invasion (Figure 3). MIP-1α was again used as a control. The IC_50 _of RANTES and AOP-RANTES in the infection assay was 0.053 nM and 0.062 nM, respectively. The IC_50 _for each inhibitor in the invasion assay was more than a log lower than the IC_50 _in the erythrocyte binding assay. The difference in response as determined by a two-way ANOVA test was significant between RANTES and MIP-1α and between AOP-RANTES and MIP-1α, but not between RANTES and AOP-RANTES (p < 0.05).

### The V3-like peptide is necessary, but not sufficient for DARC binding

The pHVDR22 plasmid expresses region II of the *P. vivax *DBP and binds to DARC+ erythrocytes when expressed on the surface of COS-7 cells. Region II includes 12 conserved cysteine residues, C1-C12, and the 32 amino acid V3-like peptide spans C1-C4. Deletion mutants lacking the V3-like peptide or adjacent sequences were made from pHVDR22 and tested for their ability to bind to DARC+ erythrocytes when expressed on COS-7 cells. The expression of each construct on the surface of COS-7 cells was confirmed by immunofluorescence staining, and the number of COS-7 cells stained was 5-10% of the population. The same number of COS-7 cells transfected with the parental pHVDR22 plasmid were stained and visualized by immunofluourescence.

**Figure 4:**
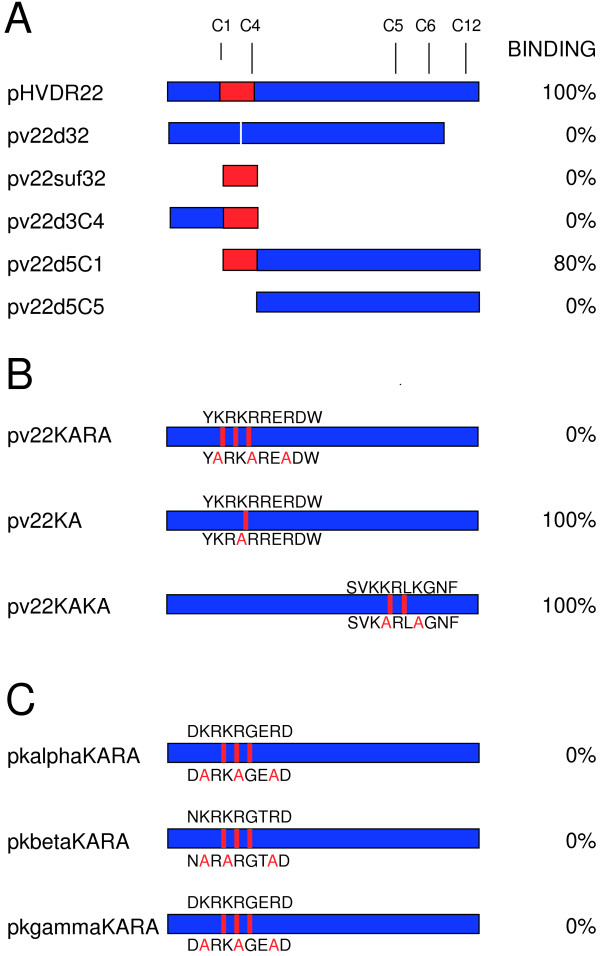
**Mutants of the region II of Erythrocyte Binding Proteins**. Panel A. *P. vivax *DBP region II is shown in blue with conserved cysteines C1, C4, C5, C6 and C12 shown. Deletion mutants in the are shown with the V3-like peptide (amino acids 216-247, between C1-C4) highlighted in red. Primers flanking this site, facing outward, were used to create pv22d32 (delete 32 amino acids) by inverse PCR. The other mutants were created with primers facing inward and containing restriction enzyme sites. Percent binding is expressed as number of rosettes compared to pHVDR22. Panel B. Site directed mutagenesis using the Stratagene QuickChange kit was used to make alanine substitutions within the consensus heparin binding site of the V3-like peptide (R22KARA, R22KA), or another consensus site (R22KAKA) at amino acids 364-373, between conserved cysteines C5-C6. Panel C. Site directed mutagenesis was used to created the same KARA mutation in the conserved heparin binding site between C1-C4 of the *P. knowlesi *α, β and γ proteins.

The pv22d32 construct that specifically deleted the V3-like peptide completely failed to bind to DARC+ erythrocytes in the erythrocyte binding assay (Figure 4A). This suggests that the DBP V3-like peptide is necessary for DARC binding. The deletion of all flanking sequences to the V3-like peptide, accomplished in the pv22suf32 mutant, also abrogated binding, showing that the DBP V3-like peptide is not sufficient for DARC binding. This confirms that there are other areas of region II necessary for binding. Truncation of the amino acids flanking the DBP V3-like peptide toward the amino terminus, as accomplished in the pv22d5C construct, had only a small effect on binding. However, truncation of the region flanking the DBP V3-like peptide to the carboxy terminus, in pv22d3C4, abrogated binding. This suggests that essential binding residues are located in the C-terminal, but not the N-terminal regions flanking the DBP V3-like peptide. To confirm the need for the DBP V3-like peptide in addition to the C-terminal flanking region, truncation of the amino-terminal end of region II up to and including the DBP V3-like peptide, in construct pv22d5C4, again abrogated binding.

### A polycation sequence within the DBP V3-like Peptide is necessary for DARC Binding

To determine if the polycation sequence in the DBP is necessary for DARC binding, site directed mutagenesis was used to introduce alanine substitutions for three positively charged amino acids at K221, R224, and R227. The pv22KARA mutant contains these substitutions and does not bind DARC+ erythrocytes when expressed on COS-7 cells (Figure 4B). The six positively charged amino acids at this site of PvRII create several possible consensus heparin binding sequences of the patterns BBXB or BBBXXB, where B is a basic amino acid and × is any amino acid, including a basic one. To determine how sensitive binding is to loss of charge at this the site, a single alanine substitution was made at K223 in the pv22KA construct. This mutant was capable of binding to DARC+ erythrocytes as well as the wild type pHVDR22 protein.

One other site in PvRII, at amino acids 364-373, between C5 and C6, contains a polycationic site which conforms to a consensus heparin binding sequence. The pv22KAKA construct introduces two alanine substitutions for lysine residues in this second consensus heparin binding site at K367 and K370. The DBP region II expressed from this construct is able to bind to DARC+ erythrocytes on the surface of COS-7 cells as well as wild type pHVDR22.

### The polycationic site has a conserved Role in the DBP protein family for binding to Diverse Receptors

Studies by Ranjan and Chitnis have identified a site in PvRII in the C-terminal flanking region to the DBP V3-like peptide, between C4-C7, that contain residues necessary for DARC binding [8]. This study also showed that the C1-C4 region of the *P. knowlesi *β protein, a member of the DBP family that does not bind to DARC, was capable of substituting for the *P. vivax *C1-C4. Upon closer inspection, the polycationic site is well conserved in the DBP family, with great similarity between proteins that bind different receptors (Figure 4C). The *P. knowlesi *α and γ proteins have an identical consensus heparin binding site, but only a binds to DARC. To see if the polycationic site may play a similar role in the binding proteins of other members of the DBP family, the same three alanine substitutions found in pv22KARA were introduced by site directed mutagenesis into the plasmids pHKADR22, pHKBDR22, and pHKGDR22. This yielded the constructs pkalphaKARA, pkbetaKARA, and pkgammaKARA, which contain the K221, R224, and R227 alanine substitution in the *P. knowlesi *α, β, and γ genes, respectively. All three of these mutants failed to bind rhesus erythrocytes when expressed in COS-7 cells.

## Discussion

HIV-1 binds to chemokine receptors such as CCR5 and CXCR4 using SU, and can be inhibited from *in vivo *infection by mutation of the chemokine receptors or by incubation with chemokines, such as RANTES. Likewise, *P. vivax *uses its DBP to bind to DARC and can be inhibited by null mutations in the receptor or *in vitro *by MGSA or IL-8. Here, we show that the chemokine, RANTES, and its analog AOP-RANTES, known to block HIV-1 SU binding to CCR5, also blocks *P. vivax *DBP binding to DARC. This demonstrates that natural and designed chemokine inhibitors can be cross-protective to both pathogens, and may have important implications for drug and vaccine development in co-endemic areas.

The overlap in chemokine inhibition of both HIV-1 and *Plasmodium *infection supports a hypothesis that SU and PvDBP have convergently evolved to mimic chemokines in such a way that the two proteins have structural similarities. The N-terminus extracellular domain of DARC is involved in binding to both region II of the PvDBP and to chemokines, just as the N-terminus of CCR5 is critical for SU and chemokine binding. In particular, negatively charged and sulfotyrosine residues in the CCR5 N-terminus and CXCR4 extracellular domain have important interactions with the C4/V3 stem of SU, and positively charged residues are implied to be important components of the SU chemokine receptor binding surface. Similarly, Pv-DBL or Pka-DBL have important interactions with sulphated tyrosine (Tyr 41) residue on DARC [9] The results of a homology search identifying an amino-acid sequence similarity between the V3 loop of HIV-1 strain MN and a 32-aa site within region II of PvDBP containing a polycationic site (Figure 1). Other members of the EBP family share this homology or "V3-like peptide". The crystal structure of the P. knowlesi DBL domain (Pkα-DBL), which binds to DARC during infection of human erythrocytes, shows that this structure is indeed similar, with disulfide bridges between C1 and C4 and between C2 and C3 forming a random coil structure designated domain 1 [10].

To investigate the role of the V3-like peptide in DARC binding we used an established erythrocyte binding assay and made mutants of the region II PvDBP expression vector. Deletion of the 32-aa V3-like peptide in construct pv22d32, or deletion of the flanking regions in construct pv22suf32, abrogated binding to DARC, suggesting that the V3-like peptide was necessary but not sufficient for binding (Figure 4A). In particular, the region between the conserved cysteines C4-C12 was necessary for binding as demonstrated by binding of pv22d5C1 and nonbinding of pv22d3C4, but the C4-C12 region was also not sufficient as shown by nonbinding of pv22d5C4. It is possible that the deletions we have made change the folding of the receptor binding site, with the exception of the deletion of amino acids 198-216. Previous work by Ranjan and Chitnis [8] using chimeras of region II between the *P. vivax *DBP and *P. knowlesi β *protein, which does not bind DARC but sialic acid, revealed the entire C4-C7 region of PvDBP region II is necessary for DARC binding, which our data corroborate. Of note, their data show that region C4-C7 is sufficient for binding, but this does not mean that other regions are not involved binding of the full-length protein. The authors of the chimeric data suggest that residues outside of C4-C7 influence the fine specificity of the DBL binding domain [8]. This might be comparable to the specificity of chemokine receptor binding attributed to small changes in the V3 loop, which mimics the β hairpin structure in chemokines, while conserved portions of the SU molecule create the structural backbone of the chemokine receptor binding surface [11].

The polycationic site within the V3-like peptide is conserved in the EBP family and contains consensus heparin binding sequences of the patterns BBXB or BBBXXB, where B is a basic amino acid and × is any amino acid, including a basic one. It was previously shown that polyanions inhibit DARC-binding by the *P. knowlesi α *protein, and we have determined that this also to be true of the PvDBP region II (Bolton *et al.*, in preparation). We made site-directed mutations to substitute alanines for positively charged amino acids at K221, R224, and R227. This mutant, designated pv22KARA, did not bind. Such minor changes make it less likely that this mutant does not bind due to folding error than to contributions these residues make directly to receptor binding. To determimne if the polycationic site was sensitive to a single alanine substitution, we created a mutation at K223 which did not change binding in pv22KA. This mutant protein still contained consensus heparin binding sequences and five positively charged residues at the site. We also mutated the only other polycationic site in the PvDBP region II that conforms to a consensus heparin binding sequence at amino acids 364-373 by substituting alanines at K367 and K370. pv22KAKA was still able to bind. These data show that the polycation site in the V3-like peptide is discretely involved in DARC binding and suggest the multiple positive charges play a redundant role at the site.

**Figure 5:**
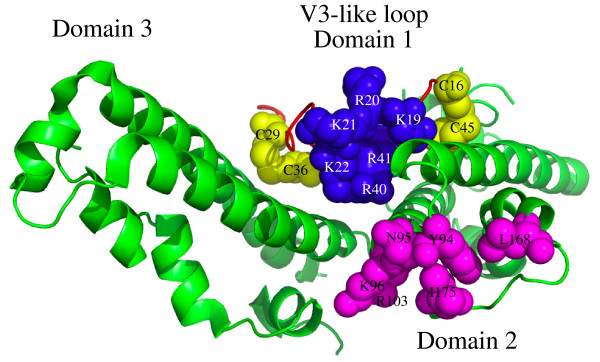
**Three dimensional location of heparin binding motif in relation to known binding residues on the crystal structure of recombinant Pkα-DBL**. The crystal structure of the recombinant Pkα-DBL that binds to human DARC is shown with previously described binding residues Tyr 94, Asn 95, Lys 96, Arg 103, Leu 168 and Ile 175 [10,12-14] highlighted in magenta in domain 2 of the molecule. The heparin binding motif on the V3-like peptide is highlighted in yellow cysteines C1-C4 and blue for the basic residues (lysine and arginine) in domain 1 of the molecule.

Previous site-directed mutagenesis experiments have identified residues Tyr 94, Asn 95, Lys 96, Arg 103, Leu 168 and Ile 175 on domain 2 as required for recognition of DARC on human erythrocytes [12-14]. Based on the crystal structure of the Pkα-DBL these residues lie close to a set of positively charged residues Lys 96, Lys 100, Arg 103 and Lys 177 that have been suggested to interact with the sulphate group on DARC Tyr 41 [10]. Mapping the polycationic site we found to be sensitive to alanine substituions onto the crystal structure shows that it is adjacent to the putative binding site residues and may provide such an interaction with the sulphated Tyr 41 (Figure 5).

The *P. knowlesi α*, β, and γ proteins share the V3-like loop and polycation site homology in region II with PvDBP, though only the α protein binds DARC. We introduced similar alanine mutations into three positively-charged amino acids of each of the three *P. knowlesi *EBPs at the polycationic site. In all 3 cases this eliminated normal binding to rhesus erythrocytes. In the case of the *P. knowlesi α *protein this reinforces the conclusion that the site can contribute to DARC binding. The *P. knowlesi *β, and γ proteins, however, don't bind to DARC. The receptor for the *P. knowlesi *β protein is sialic acid, which is negatively charged for which the polycation site might contribute to a positively charged binding pocket. A chimera produced by Ranjan and Chitnis [8] with the C1-C4 region exchanged between the *P. knowlesi β *protein and *P. vivax *DBP bound to DARC on rhesus erythrocytes, without the removal of sialic acid residues required for native *P. vivax *DBP to bind to rhesus DARC. It is possible that a closer homology between the polycationic site of *P. knowlesi α *and β proteins, each of which contain 5 cationic residues versus the 6 cationic residues of the *P. vivax *DBP polycationic site, allows for this change in specificity. Another chimera in the same study with the *P. vivax *polycationic site is able to bind to rhesus erythrocytes in the same manner as the *P. knowlesi β *protein, but only in the presence of C4-C5 of the *P. knowlesi β *protein. This again suggests that the homology of the polycationic site within the EBP family may allow for a redundant function in receptor binding, but the role of the polycationic site is in conjunction with other residues in region II which together allow for efficient receptor binding. The results presented here, in conjunction with previous studies, indicate that the heparin binding site motif in members of the DBP family may form part of a conserved erythrocyte receptor binding pocket.

## Competing interests

The authors declare that they have no competing interests.

## Authors' contributions

MJB performed the investigations described in this study. MJB and RFG conceived of the study, and RFG participated in its design and coordination and helped to draft the manuscript. Both authors read and approved the final manuscript.
